# Assessment of Management Options on Striga Infestation and Maize Grain Yield in Kenya

**DOI:** 10.1017/wsc.2018.4

**Published:** 2018-07-01

**Authors:** Fred Kanampiu, Dan Makumbi, Edna Mageto, Gospel Omanya, Sammy Waruingi, Peter Musyoka, Joel Ransom

**Affiliations:** 1 Senior Scientist, International Institute of Tropical Agriculture, Nairobi, Kenya; 2 Senior Scientist, International Maize and Wheat Improvement Center, Nairobi, Kenya; 3 Graduate Student, Department of Agronomy, Iowa State University, Ames, IA 50011, USA; 4 Senior Project Manager, African Agricultural Technology Foundation, Nairobi, Kenya; 5 Managing Director, DUDU East Africa Ltd, Thika, Kenya; 6 Project Officer, African Agricultural Technology Foundation, Nairobi, Kenya and; 7 Professor and Extension Agronomist, North Dakota State University, Department of Plant Sciences, Fargo, ND 58108, USA

**Keywords:** Cropping systems, herbicide resistance, imazapyr-resistant hybrids, legumes, maize varieties, peanut

## Abstract

The parasitic purple witchweed [Striga hermonthica (Del.) Benth.] is a serious constraint to maize production in sub-Saharan Africa, especially in poor soils. Various Striga spp. control measures have been developed, but these have not been assessed in an integrated system. This study was conducted to evaluate a set of promising technologies for S. hermonthica management in western Kenya. We evaluated three maize genotypes either intercropped with peanut (Arachis hypogaea L.), soybean [Glycine max (L.) Merr.], or silverleaf desmodium [Desmodium uncinatum (Jacq.) DC] or as a sole crop at two locations under artificial S. hermonthica infestation and at three locations under natural S. hermonthica infestation between 2011 and 2013. Combined ANOVA showed significant (P < 0.05) cropping system and cropping system by environment interactions for most traits measured. Grain yield was highest for maize grown in soybean rotation (3,672 kg ha−1) under artificial infestation and in D. uncinatum and peanut cropping systems (3,203 kg ha−1 and 3,193 kg ha−1) under natural infestation. Grain yield was highest for the Striga spp.-resistant hybrid under both methods of infestation. A lower number of emerged S. hermonthica plants per square meter were recorded at 10 and 12 wk after planting on maize grown under D. uncinatum in the artificial S. hermonthica infestation. A combination of herbicide-resistant maize varieties intercropped with legumes was a more effective method for S. hermonthica control than individual component technologies. Herbicide-resistant and Striga spp.-resistant maize integrated with legumes would help reduce the Striga spp. seedbank in the soil. Farmers should be encouraged to adopt an integrated approach to control Striga spp. for better maize yields.

## Introduction

In sub-Saharan Africa (SSA) the parasitic weeds in the genus Striga are a serious constraint to the productivity of staple cereal crops such as maize (*Zea mays* L.), sorghum [*Sorghum bicolor* (L.) Moench.], pearl millet [*Pennisetum glaucum* (L.) R. Br.], and upland rice (*Oryza sativa* L.) (Ejeta 2007; Oswald and Ransom 2001). The most important among the Striga species in Africa are purple witchweed [Striga hermonthica (Del.) Benth.] and Asiatic witchweed [Striga asiatica (L.) Kuntze] (Gethi et al. 2005; Gressel et al. 2004). Striga spp. survive by siphoning off water and nutrients from the host crop for its own growth and exerts a potent phytotoxic effect. It impairs normal host-plant growth, resulting in a large reduction in plant height, biomass, and eventual grain yield (Gurney et al. 1995). Striga spp. infestation is most severe in areas with low soil fertility and low rainfall and in farming systems characterized by intensive cultivation with poor crop management and less use of inputs such as fertilizer, pesticides, and improved seeds (Ransom 2000). Striga spp. infests nearly 100 million ha in SSA (Lagoke et al. 1991) and causes yield losses ranging from 20% to 80% and even total crop failure in cases of severe infestation (Ejeta et al. 2007; Kanampiu et al. 2002; Khan et al. 2006). Despite the increased yield in some countries due to use of improved seeds and fertilizers, the yield of maize when Striga spp. is present is as low as 1,000 kg ha−1, displaying some of the lowest yields in the world (Cairns et al. 2013).

The *S. hermonthica* problem has persisted with limited adoption of recommended control methods owing to farmers’ reluctance to adopt such methods and unfavorable biological and socioeconomic conditions (Kanampiu et al. 2003; Oswald 2005). Effective S. hermonthica control technologies should target reducing the seedbank, limiting the production of new seeds and their spread from infested to noninfested soils, improving soil fertility and methods that fit within the farmers’ cropping system, all of which should result in good crop yield (Ejeta 2007; Khan et al. 2006).

Relatively good progress in identifying S. hermonthica tolerance/ resistance in maize has been achieved (Kim 1994), and S. hermonthica-resistant (STR) varieties are being adopted in West Africa (Badu-Apraku and Lum 2007; Menkir et al. 2010, 2012a). These hybrids sustain fewer symptoms of damage and lower yield losses under S. hermonthica infestation and support fewer emerged parasites than a susceptible hybrid check (Menkir et al. 2007). A number of herbicides are available for controlling preflowering Striga spp., but they are largely unavailable to smallholder farmers, mainly because of cost. S. hermonthica infestation can be controlled by applying imazapyr to imazapyr-resistant maize (IR maize) (Kanampiu et al. 2001, 2002, 2003). Seed-dressing of IR maize allows direct action on S. hermonthica seeds that are near the maize. When S. hermonthica plants attach themselves to the maize roots near coated seeds, they immediately die. Imazapyr that is not taken up by the maize seedlings diffuses into the surrounding soil and is absorbed by ungerminated dormant S. hermonthica seeds, killing them when they germinate upon stimulation. The maize remains S. hermonthica free for the first weeks after planting, and this considerably increases yield (Kanampiu et al. 2002, 2003). This technology depletes the S. hermonthica seedbank so that subsequent S. hermonthica numbers are lower the following year; it is cost-effective and compatible with existing cropping systems. Sensitive crops like common bean (Phaseolus vulgaris L.) are unaffected when intercropped but planted at >15 cm from maize coated with imazapyr herbicide (Kanampiu et al. 2002). Therefore, simple herbicide seed coatings are thus compatible with commonly used African intercropping systems, while facilitating maize growth and depleting the Striga spp. seedbank.

Hand weeding seems to be a straightforward approach to interrupt the growth cycle of S. hermonthica and is easy to practice and understand. Nevertheless, it is not very effective, and farmers are reluctant to employ it, since S. hermonthica plants become large enough to be uprooted only after the first weeding for the crop (Oswald 2005), and it is extremely time-consuming at high densities. Nevertheless, hand weeding remains an integral part of an integrated Striga spp. control approach to minimize mature plants and diminish the seedbank.

Intercropping of cereals with legumes such as cowpea [Vigna unguiculata (L.) Walp.], peanut (also known as groundnut), mungbean (Vigna radiata L.), bonavist-bean (Dolichos lablab L.), and soybean, has been shown to reduce the number of Striga spp. plants that mature in an infested field (Carsky et al. 1994). Intercrops can act as trap crops, stimulating suicidal Striga spp. germination or altering the microclimate of the crop’s canopy and soil surface to interfere with Striga spp. germination and development (Parker and Riches 1993). This push‒pull technology has been used to effectively manage S. hermonthica in sorghum and maize-based cropping systems where maize is intercropped with a stem borer–repellent plant, D. uncinatum, and an attractant host plant, Napiergrass (Pennisetum purpureum Schumach.), is planted as a trap plant around the field (Khan et al. 2006). Though it is novel, adoption of the push–pull system is constrained, because Desmodium spp. is a fodder crop that cannot be directly used as food (Odhiambo et al. 2011). Rotating susceptible cereal crops with trap crops such as cotton (Gossypium hirsutum L.), cowpeas, and soybean that induce the germination of S. hermonthica but are not parasitized, effectively reduces the levels of S. hermonthica seed in the soil (Ariga 1996). However, the choice of crops used in rotations should be based on their adaptability to agroecological conditions, ability to reduce the Striga spp. seedbank, contribution to improving soil fertility (nitrogen fixation, production of high amounts of organic matter, etc.), productivity, and marketability. Soybean induces germination of S. hermonthica but is not parasitized (Carsky et al. 1998; Odhiambo et al. 2011; Sanginga et al. 2003) making the legume a good choice for crop rotation in Striga spp. management.

Different Striga spp. management strategies have been proposed and individually evaluated, but these strategies or their combinations have not been evaluated in integrated trials. The objective of this study was to evaluate a set of promising integrated technologies for S. hermonthica control in western Kenya. The main hypothesis was that integration of technologies that exist can elucidate a better response in S. hermonthica control and increase maize production in the affected target areas. This multilocational and multiseasonal evaluation was implemented through on-station and on-farm trials.

## Materials and Methods

### Plant Materials

This study used maize, peanut (groundnut), soybean, and D. uncinatum.

### Maize

Three types of maize varieties with different characteristics (herbicide resistance, S. hermonthica resistance, and susceptible) were used in this study. Two herbicide-resistant varieties included in the trial were a commercially available IR hybrid ‘Ua Kayongo I’ and ‘WS303,’ an open-pollinated variety (OPV). Both are among the first-generation of IR maize varieties developed by the International Maize and Wheat Improvement Center (CIMMYT). The development of IR maize varieties was described in detail by Makumbi et al. (2015). The S. hermonthica-resistant maize hybrid H12 (‘0804-7STR’), which was developed by the International Institute of Tropical Agriculture (IITA), was also included in this study. This hybrid had a very good grain yield and resistance to foliar diseases under S. hermonthica infestation in trials conducted in Kenya and Nigeria (Menkir et al. 2012b). The commercial CIMMYT-derived hybrid maize variety, ‘WH403,’ was selected as a susceptible check.

### Peanut

The variety ‘ICGV 907048SM,’ grown widely in western Kenya, was used. It is high yielding with a high oil content and resistant to peanut rosette disease. Seed was obtained from the International Center for Tropical Agriculture (CIAT).

### Soybean

The variety ‘TGx1740-2F’ (‘SB 19’) is grown widely in western Kenya. It is one of the best-adapted varieties with a high potential for biological nitrogen fixation, high yield, and good market value; it has also been shown to significantly induce suicidal S. hermonthica germination (Omondi et al. 2014). The seed was obtained from CIAT.

### Desmodium uncinatum

Seeds of D. uncinatum used in a previous study (Khan et al. 2006) were obtained from the International Centre of Insect Physiology and Ecology.

### Treatments and Experimental Design

Treatments consisted of a factorial combination of maize genotypes and cropping systems. The whole-plot factor (cropping system) included the following levels: D. uncinatum, peanut, maize only, and maize rotation with soybean. The subplot factor (maize genotype) consisted of the following levels: IR hybrid, IR OPV, STR hybrid, and WH403. The IR maize seed was coated with 0.56 mg imazapyr seed−1 (30 g imazapyr ha−1), as described in detail by Kanampiu et al. (2003).

The whole-plot treatments were laid out in a randomized complete block design with three replications. Each experimental unit consisted of 4 rows, 5-m long, at a spacing of 75 by 50 cm, and 3 seeds hill−1, later thinned to 2 plants hill−1 to give a plant population of 53,333 ha−1, for each management option of maize. Peanut was planted at a spacing of 15 by 5cm between maize rows, 1 plant hill−1, to give a plant population of 533,333 ha−1. D. uncinatum was drilled in the middle of two maize rows. Soybean was drilled in rows, 75-cm apart, and thinned to 5 cm between plants at about 2 wk after germination to give a plant population of 266,666 ha−1 as a sole crop after every other season.

## Field Protocols

### On-Station Trials

On-station trials at Kibos and Alupe were artificially infested with S. hermonthica seeds.

S. hermonthica seeds were added to each planting hole to ensure that each maize plant was exposed to a minimum of 2,000 viable S. hermonthica seeds. About 8,000 S. hermonthica seeds collected from Kibos Station fields, containing about 25% extraneous material and 70% viability in 10 g of soil/seed mixture, were added to an enlarged planting hole at a depth of 7 to 10 cm directly below the maize.

Weeding was done by hand to remove all weeds except S. hermonthica from the field. D. uncinatum plants were kept in the plots at the end of each season, owing to their perennial nature, and trimmed down to allow maize to be planted in between rows for the subsequent seasons. In the soybean‒maize rotation cropping system, plots were initially cropped with soybean alone followed by mono-cropped maize the following season.

**On-station trials.** Five locations were used to evaluate the trials under on-station and on-farm conditions in western Kenya. Kibos (−0.03861°S, 34.81596°E; 1,193 m above sea level (masl); 865 mm mean annual rainfall bimodal distribution) and Alupe (0.503725°N, 34.12148°E; 1,153 masl; 1,400 mm mean annual rainfall bimodal distribution) were used for on-station trials. These two locations are routinely used for screening maize under artificial S. hermonthica infestation in Kenya. The trials were evaluated during the long rainy seasons (March to August) in 2012 and 2013 (2012A and 2013A) and short rainy seasons (October to February) in 2011 and 2012 (2011B and 2012B) at both locations.

### On-Farm Trials

Natural infestation sites were selected from farmer’s fields that were historically known to be highly S. hermonthica infested. No S. hermonthica seed was added to the on-farm field trials. The on farm trials were researcher managed with assistance from the hosting farmers. Farmers applied fertilizers and weeded plots with a hand hoe, based on guidance from the researchers. Weeding was done 3 wk after planting (WAP), and thereafter hand pulling was done to remove weeds other than S. hermonthica. Crop management was standardized for all locations. Diammonium and phosphate (18–46–0) was applied during planting at a rate of 50 kg N and 128 kg P2O5 ha−1, and top-dressing was done at 6 WAP with calcium ammonium nitrate at a rate of 50 kg N ha−1.

**On-farm trials.** Three sites, namely Teso (0.478092°N, 34.12516°E; 1,199 masl), Siaya (0.236652°N, 34.16691°E; 1,244 masl),and Rachuonyo (−0.42738°S, 34.70928°E; 1,316 masl) were used for evaluation under on-farm conditions. These three locations were chosen for on-farm experimentation based on the uniformity and intensity of S. hermonthica infestation observed on the fields during a site-selection exercise in 2011. The trials were evaluated during the same seasons as the on-station trials. These locations were chosen because of the bimodal rainfall distribution with a long rainy season from March to July and a short rainy season from September to December, both of which are suitable for maize production.

### Data Collection

The number of emerged S. hermonthica plants was recorded at 8, 10, and 12 WAP. The total number of S. hermonthica plants per plot was calculated and expressed as S. hermonthica plants per square meter. For maize, the following were measured: days to anthesis (days from planting to when 50% of the plants had shed pollen) and plant height (measured in centimeters as the distance from the base of the plant to the height of the first tassel branch). At harvest, cobs were handpicked excluding those from plants at end of rows and weighed. A representative sample of ears was shelled to determine percentage grain moisture. Grain yield (t ha−1) was calculated from ear weight and grain moisture, assuming a shelling percentage of 80% and adjusted to 12.5% grain moisture content.

### Statistical Analysis

Because variances of S. hermonthica counts tend to be heterogeneous, they were transformed before statistical analysis using the expression Y=log(X+1), where X is the original S. hermonthica count. ANOVA across environments was performed using PROC GLM in SAS (SAS Institute 2011) for grain yield, agronomic traits, and transformed S. hermonthica count data. All treatment factors were considered fixed. The linear-effects model for a balanced split-plot design was used for ANOVA (Lentner and Bishop 1986) and is shown below:

$$Y_{ijk}={\text{μ+ρ}}_{i}+{\text{α}}_{j}+{\text{δ}}_{ij}+{\text{β}}_{k}+{\left(\text{αβ}\right)}_{jk}+{\text{ε}}_{ijk}$$

where $Y_{ijk}$ is the observed mean for a genotype; μ is the overall mean a constant; ρ_i_ is the effect due to the ith replicate; α_j_ is the effect of the jth level of A (whole-plot); δ_ij_ is whole-plot error component; βk is the effect of the kth level of B (subplot); ${\left(\text{αβ}\right)}_{jk}$ is the interaction effect of the jth level of A and the kth level of B; and ${\text{ε}}_{ijk}$ is the split-plot error. The assumptions for this model are that δ values are independent identically distributed (i.i.d.) $N\left(0,\sigma^{2}{}_{\delta }\right)$, ε values are i.i.d$N\left(0,\sigma^{2}{}_{\varepsilon }\right)$, and δ and ε values are distributed independently of each other. A combination of season by location was considered an environment. Tukey’s HSD test was used for pair-wise mean comparison. Pearson correlation coefficients between traits were calculated using PROC CORR procedure in SAS (SAS Institute 2011).

To assess consistency of management combinations under artificial and natural S. hermonthica infestation, Kendall’s (1970) coefficient of concordance (W statistic) was computed for each trait based on ranks of entry means recorded across on-station and on-farm and across locations. Kendall’s W statistic is obtained as:

$$W=\frac{12S}{p^{2}\left(n^{3}-n\right)-pT}$$( 1 )

in which S is a sum-of-squares statistic over the row sums of ranks, n is the number of treatment combinations, p is the number of locations, and T is a correction factor for tied ranks.

## Results and Discussion

### Effect of Cropping System and Variety on Agronomic Traits and S. hermonthica Emergence

### Cropping System

**Figure 1:**
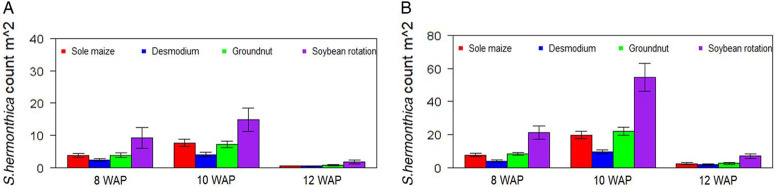
Mean number of emerged S. hermonthica plants at 8, 10, and 12 wk after planting (WAP) for four cropping systems under artificial (A) and natural (B) S. hermonthica infestation for 3 yr (2011–2013). The whiskers represent SEs.

There was a significant effect of cropping system (P < 0.05) for all traits measured under artificial S. hermonthica infestation (Table 1). However, under natural infestation, the cropping system had a significant effect (P < 0.05) only on S. hermonthica plant emergence and grain yield (Table 2). The cropping system by environment interaction was significant only for S. hermonthica count at 8 WAP under artificial S. hermonthica infestation (Table 1) and for the three S. hermonthica count parameters under natural S. hermonthica infestation (Table 2). Under artificial S. hermonthica infestation, maize hybrids exhibited earlier flowering in the soybean rotation. The maize was shorter when grown under D. uncinatum and in the maize-only crop than in other cropping systems. Grain yield was highest for maize grown in the soybean rotation (3,672 kg ha−1) followed by intercropping with peanut (3,575 kg ha) (Table 3). Under natural S. hermonthica infestation, grain yield was highest for maize under D. uncinatum and peanut (3,203 kg ha−1 and 3,193 kg ha−1) cropping systems (Table 3). The number of emerged S. hermonthica plants m−2 was lowest on maize grown in D. uncinatum, peanut, and maize-only cropping systems at 8 WAP, and lowest on maize under D. uncinatum at 10 and 12 WAP under artificial S. hermonthica infestation (Figure 1). The number of emerged S. hermonthica plants m−2 at 8, 10, and 12 WAP was highest in maize grown in plots under soybean rotation under both artificial and natural S. hermonthica infestation (Figure 1).

**Table 1:** Mean squares from combined ANOVA for agronomic traits and S. hermonthica counts under artificial S. hermonthica infestation at two locations (Kibos and Alupe) in Kenya across 3 yr (2011–2013).^a^

Source	df	AD	df	PH	STR8	STR10	STR12	df	GY
Env	7	1,441.97^***^	4	12,250.26^***^	1.16^***^	6.24^***^	10.74^***^	6	28,438,114.6^***^
Rep (Env)	14	39.58^*^	8	2,085.56^***^	0.15ns	0.33^*^	0.54^***^	12	4,137,463.0^***^
CS^b^	3	59.96^*^	3	2,171.38^*^	0.62^*^	1.89^***^	2.56^***^	3	5,835,583.6^*^
Variety^c^	3	634.31^***^	3	4,500.83^***^	19.10^***^	26.01^***^	17.27^***^	3	26,797,512.4^***^
CS × Variety	9	11.09^*^	9	217.22ns	0.21ns	0.24ns	0.16ns	9	991,523.4ns
CS × Env	17	14.82ns	10	1,354.48ns	0.31^*^	0.30ns	0.28ns	15	2,827,053.0ns
Variety × Env	21	64.68^***^	12	876.25^***^	0.40^***^	1.28^***^	1.41^***^	18	2,690,252.0^***^
CS × Variety × Env	51	7.64ns	30	221.41ns	0.18ns	0.33^**^	0.20ns	45	538,504.5ns
CS × Rep (Env)	40	13.97^***^	26	628.53^***^	0.16ns	0.18ns	0.18ns	36	1,734,084.4^***^
Variety × Rep (Env)	48	5.13ns	30	236.02ns	0.10ns	0.14ns	0.13ns	42	470,275.4ns
Error	120	5.32	78	173.54	0.14	0.18	0.14	108	659,890.0

^a^Abbreviations: AD, days to anthesis; CS, cropping system; Env, environment; GY, grain yield in metric tons per hectare; ns, not significant; PH, plant height in centimeters; STR8,S. hermonthica count at 8 wk after planting (WAP); STR10, S. hermonthica count at 10 WAP; STR12, S. hermonthica count at 12 WAP; Rep, replication.

^b^Cropping system (main plot): Desmodium spp.; maize only; peanut; soybean rotation.

^c^ Variety (subplot): imazpyr-resistant hybrid, imidazolinone-resistant open-pollinated variety, S. hermonthica-resistant hybrid, and commercial maize hybrid.

^*^significant at P < 0.05;

^**^significant at P < 0.01;

^***^significant at P < 0.001.

**Table 2:** Mean squares from combined ANOVA for agronomic traits and S. hermonthica counts under natural S. hermonthica infestation at three locations (Siaya, Teso, and Rachuonyo) in Kenya across 3 yr (2011–2013).^a^

Source	df	AD	df	PH	STR8	STR10	STR12	df	GY
Env	6	5,463.46^***^	10	177,452.01^***^	9.55^***^	10.91^***^	12.05^***^	11	73,561,236.6^***^
Rep (Env)	12	5.16^**^	20	1,156.58^***^	0.38^***^	0.49^***^	0.25^***^	22	2,140,330.6^***^
CS^b^	3	5.17ns	3	518.22ns	2.86^***^	8.55^***^	7.02^***^	3	4,706,516.5^*^
Variety^c^	3	156.63^***^	3	1,707.97^**^	23.19^***^	17.05^***^	12.38^***^	3	25,490,761.9^***^
CS × Variety	9	0.56ns	9	720.12^*^	0.19^*^	0.09ns	0.08ns	9	2,163,813.0^***^
CS × Env	15	4.93ns	25	719.27ns	0.87^***^	0.71^***^	0.66^***^	27	1,551,125.4ns
Variety × Env	18	53.43^***^	30	1,591.18^***^	1.88^***^	1.42^***^	1.48^***^	33	4,821,431.3^***^
CS × Variety × Env	45	2.09ns	75	352.47ns	0.11ns	0.13ns	0.08ns	81	760,160.4^***^
CS × Rep (Env)	36	3.74^**^	56	590.99^***^	0.21^***^	0.23^**^	0.21^***^	60	1,167,614.2^**^*
Variety × Rep (Env)	42	2.23ns	66	330.11ns	0.17^***^	0.11ns	0.09ns	72	442,532.4ns
Error	106	1.98	168	286.63	0.09	0.13	0.08	180	378,988

a Abbreviations: AD, days to anthesis; CS, cropping system; Env, environment; GY, grain yield in metric tons per hectare; ns, not significant; PH, plant height in centimeters; STR8,S. hermonthica count at 8 wk after planting (WAP); STR10, S. hermonthica count at 10 WAP; STR12, S. hermonthica count at 12 WAP.

b Cropping system (main plot): Desmodium spp.; maize only; peanut; soybean rotation.

c Variety (subplot): imazpyr-resistant hybrid, imidazolinone-resistant open-pollinated variety, S. hermonthica-resistant hybrid, and commercial maize hybrid.

* significant at P < 0.05;

** significant at P < 0.01;

*** significant at P < 0.001.

**Table 3:** Mean grain yield and other agronomic traits for different treatment combinations under artificial and natural S. hermonthica infestation at five locations in Kenya across 3 yr (2011–2013).

		Artificial infestation			Natural infestation		
Main plot	Subplot^c^	Days to anthesis	Plant height	Grain yield	Days to anthesis	Plant height	Grain yield
			–cm–	–kg ha^−1^–		–cm–	–kg ha^−1^–
Mean (main plot)^a^	Desmodium	74	189	2,948	73	163	3,203
	Peanut	73	200	3,575	72	163	3,193
	Maize only	74	190	3,140	72	160	2,847
	Soybean rotation	70	201	3,672	74	174	2,912
	Minimum SD	1	7	356	1	6	210
Mean (subplot)^b^	IR HYB maize	77	189	2,340	75	161	2,339
	IR OPV maize	75	195	3,319	73	164	3,059
	STR hybrid maize	71	207	3,873	72	168	3,542
	WH403	70	186	3,639	71	161	3,287
	Minimum SD	1	7	346	1	6	201
	CV (%)	3.15	6.78	24.7	1.94	10.35	20.2

a Cropping system (main plot): Desmodium spp.; maize only; peanut; soybean rotation.

b Variety (subplot): IR HYB, imazpyr-resistant hybrid; IR OPV, imidazolinone-resistant open-pollinated variety; STR HYB, S. hermonthica-resistant hybrid; WH403, commercial maize hybrid.

c Abbreviations: CV, coefficient of variation; SD, significant difference.

The observed fewer days to flowering and greater maize plant height with the soybean rotation compared with other cropping systems were probably due to reduced competition for nutrients and water. Previous studies have showed soybean rotation has “residual effects” (nitrogen fixed) and contributes more benefits to maize than other cropping systems (Yusuf et al. 2009). This boosted the following maize crop under this cropping system, most likely owing to improved nutrient availability and reduced biotic pressures (Vanlauwe et al. 2000). Other cropping systems probably had competitive effects with the maize crop and therefore negatively affected the performance of maize. A previous study showed that legume intercrops reduced maize yields slightly under certain intercropping system in the study area (Oswald et al. 2002). Because soybean and peanut are edible legumes and their inclusion in the cropping system increased maize yields, we expect smallholder farmers would readily accept this system, and this would in turn be an effective method for Striga spp. management in this region. Dual purpose soybean varieties that produce leafy biomass without sacrificing high grain yields resulted in substantial yield increases for a subsequent maize crop compared with less-leafy varieties (Sanginga et al. 2003). Studies have further shown that maize growing after these improved soybean varieties had a 1.2- to 2.3-fold increase in grain yield compared with the control (Sanginga et al. 2002). Though S. hermonthica emergence increased with time across all cropping systems, D. uncinatum and peanut were most effective in later S. hermonthica control, while the soybean‒maize rotation was least effective. The maize crop under soybean rotation was probably more robust and among the best in terms of maize yield due to green manuring effect of the soybean, despite this being the least effective option for managing S. hermonthica. Rotation with soybean (non-host) crop is supposed to trigger suicidal germination of S. hermonthica (Sanginga et al. 2003), leading to a decline in seed production and a reduced seedbank over the years. Observations made in this study show more seasons of soybean rotation would be required for effective S. hermonthica control. D. uncinatum was observed to significantly reduce S. hermonthica emergence. Similar findings were observed by Vanlauwe et al. (2008). D. uncinatum reduced S. hermonthica emergence due to its ability to produce metabolites that stimulate S. hermonthica seed germination but also have postgermination inhibitory activities that interfere with parasitism (Khan et al. 2008). Furthermore, given that most smallholder farmers practice mixed cropping and keep livestock, Desmodium spp. would be a good source of protein for livestock where intercropping is an integral part of the farming system (Abate et al. 2000).

### Variety

**Figure 2:**
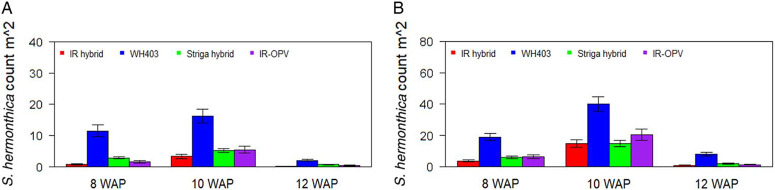
Mean number of emerged S. hermonthica plants at 8, 10, and 12 wk after planting (WAP) for four maize varieties under artificial (A) and natural (B) S. hermonthica infestation for 3 yr (2011–2013). The whiskers represent SEs. IR hybrid, imazapyr-resistant maize hybrid; IR-OPV, imazapyr-resistant maize, open-pollinated variety; Striga hybrid, S. hermonthica-resistant maize hybrid; WH403, commercial maize hybrid.

Variety and variety by environment interaction were significant for all traits under both artificial and natural S. hermonthica infestation (Table 1 and Table 2). Under both artificial and natural S. hermonthica infestation the commercial maize variety WH403 and the STR hybrid flowered earlier and had shorter plants than the other two varieties (Table 3). Grain yield was highest for the STR hybrid under both artificial (3,873 kg ha−1) and natural (3,542 kg t ha−1) infestation. The number of emerged S. hermonthica plants at 8, 10, and 12 WAP was lowest on the IR maize varieties under artificial infestation (Figure 2). The commercial hybrid WH403 supported the largest number of emerged S. hermonthica plants. Under natural S. hermonthica infestation, the IR maize hybrid supported a small number of emerged S. hermonthica plants at 8 and 10 WAP, but the STR hybrid supported a lower number at 12 WAP than both IR varieties (Figure 2).

The commercial maize hybrid WH403 and the STR hybrid flowered earlier than the other two IR varieties, probably because of stress caused by competition for water and nutrients by high S. hermonthica emergence. The hybrid WH403 had shorter plants that were associated with high S. hermonthica emergence under both artificial and natural S. hermonthica infestation. Striga spp. attachment inhibits cell elongation in meristematic cells, resulting in short internodes and stunted plants (Hood et al. 1998). Less stunting was observed for the STR hybrid due to its ability to withstand the effects of S. hermonthica infestation, and it therefore produced a higher grain yield compared with other varieties. Similar results have been reported for STR germplasm (Menkir et al. 2012b). Use of STR germplasm should benefit farmers in Striga spp.-endemic areas in this region. The IR OPV performed better than the first-generation IR top-cross hybrid, probably due to adaptation differences. The IR OPV is marketed widely in the study area, while the IR hybrid is grown in very few areas. However, new higher-yielding and better-adapted IR maize hybrids are now being developed for commercialization by several local seed companies. Therefore, we expect area coverage of these new hybrids to increase in the coming years. Reduced and late Striga spp. emergence leads to fewer plants flowering and a reduced Striga spp. seedbank with eventual reduction of infestation for crops in the following season. The results revealed consistent rankings for different management systems that suggest stable reaction patterns of the different maize varieties and S. hermonthica control options across different infestation types.

We used Kendall’s coefficient of concordance to assess the consistency of rankings of trait means for the different management combinations. The coefficient of concordance (W) was high and significant (P < 0.0001) for all traits under artificial and natural infestation and across all conditions, except for plant height under natural infestation, which was significant at P < 0.05 (Table 4). This suggested consistent rankings and stable reactions patterns of the different maize varieties under the different control options across different infestation methods. Consistent performance of maize varieties under contrasting environments has been reported in other studies by Menkir et al. (2012a, 2012b) and Makumbi et al. (2015). Consistency of rankings may also imply that S. hermonthica biotypes and/or their virulence are similar in the expansive western Kenya region where this study was conducted. This is consistent with results by Gethi et al. (2005) using amplified fragment-length polymorphism markers that revealed very little genetic diversity among 24 populations of S. hermonthica collected from western Kenya, the region where this study was conducted. The study of genetic variability in Striga spp. populations is of great importance in breeding for resistance, because it helps to target the areas and appropriate sources of resistance for identified biotypes. It is also important in designing cultivar-screening strategies, especially for the sourcing of parasite seeds. Exploiting host genetic variability to increase the level of resistance to the parasite can be a major component of an integrated approach to minimize yield losses from S. hermonthica in farmers’ fields.

**Table 4:** Kendall’s coefficient of concordance (W) for traits of maize varieties evaluated under different management options under artificial and natural S. hermonthica infestation in Kenya.

Trait^a^	Number of locations	Kendall’s coefficient, *W*	P value
Across artificial *S. hermonthica* infestation			
Grain yield	7	0.827	<0.0001
Days to anthesis	8	0.927	<0.0001
Plant height (cm)	5	0.669	0.0007
*S. hermonthica* plant count 8 WAP	8	0.828	<0.0001
*S. hermonthica* plant count 10 WAP	8	0.863	<0.0001
*S. hermonthica* plant count 12 WAP	8	0.803	<0.0001
Across natural *S. hermonthica* infestation			
Grain yield	12	0.831	<0.0001
Days to anthesis	7	0.929	<0.0001
Plant height (cm)	11	0.559	0.0165
*S. hermonthica* plant count 8 WAP	12	0.846	<0.0001
*S. hermonthica* plant count 10 WAP	12	0.848	<0.0001
*S. hermonthica* plant count 12 WAP	12	0.942	<0.0001
Across artificial and natural infestation			
Grain yield	19	0.880	<0.0001
Days to anthesis	15	0.923	<0.0001
Plant height (cm)	16	0.671	0.0007
S. hermonthica plant count 8 WAP	20	0.875	<0.0001
S. hermonthica plant count 10 WAP	20	0.888	<0.0001
S. hermonthica plant count 12 WAP	20	0.949	<0.0001

a Abbreviation: WAP, weeks after planting.

### Cropping System by Variety Interaction

**Figure 3:**
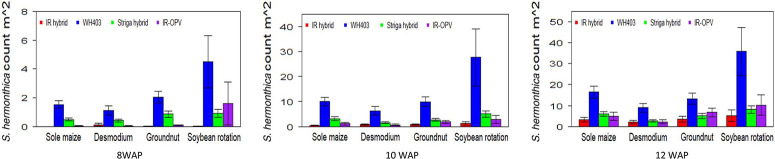
Mean S. hermonthica emergence at 8, 10, and 12 wk after planting (WAP) for four cropping systems under artificial S. hermonthica infestation at two locations for 3 yr (2011–2013). IR hybrid, imazapyr-resistant maize hybrid; IR-OPV, imazapyr-resistant maize, open-pollinated variety; S. hermonthica hybrid, S. hermonthica-resistant maize hybrid; WH403, commercial maize hybrid. The whiskers represent SEs.

**Figure 4:**
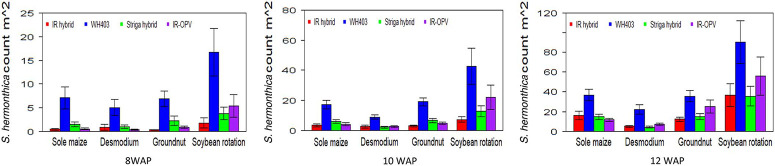
Mean S. hermonthica emergence at 8, 10, and 12 wk after planting (WAP) for four cropping systems under natural S. hermonthica infestation at three locations for 3 yr (2011–2013). IR hybrid, imazapyr-resistant maize hybrid; IR-OPV, imazapyr-resistant maize, open-pollinated variety; Striga hybrid, S. hermonthica-resistant maize hybrid; WH403, commercial maize hybrid. The whiskers represent SEs.

The cropping system by variety interaction was significant for days to anthesis under artificial *S. hermonthica* infestation and for plant height, *S. hermonthica* count at 8 WAP, and grain yield under natural infestation (Tables 1 and 2). Maize plants grown under the soybean–STR maize hybrid combination were the tallest (185 cm) followed by those under soybean–IR maize hybrid (177 cm), while the peanut–IR maize hybrid had the shortest plants under natural infestation (Table 5). Grain yield was highest for maize in the peanut–STR hybrid combination (3,829 kg ha^−1^), followed closely by the D. uncinatum–commercial maize hybrid (WH403) combination under natural infestation. The number of emerged *S. hermonthica* plants recorded was lowest for cropping system combinations involving IR maize varieties at 8, 10, and 12 WAP under both artificial and natural infestations (Figures 3 and 4). Under natural *S. hermonthica* infestation, the crop management combination with the STR hybrid was the best for control of *S. hermonthica* at 12 WAP (Figure 4).

**Table 5:** Mean grain yield and other agronomic traits for different treatment combinations under artificial and natural S. hermonthica infestation at five locations in Kenya across 3 yr (2011–2013).

		Artificial infestation			Natural infestation		
Main plot^a^	Subplot^b^,^c^	Days to anthesis	Plant height	Grain yield	Days to anthesis	Plant height	Grain yield
			–cm–	–kg ha^−1^–	75	–cm–	–kg ha^−1^–
Desmodium spp.	IR hybrid maize	77	186	2,037	75	162	2,224
	IR OPV maize	75	190	3,180	72	164	3,258
	STR hybrid maize	72	197	3,454	72	165	3,555
	WH403	72	184	3,120	71	162	3,735
	SE	1.2	2.9	312	0.9	0.8	337
Peanut	IR hybrid maize	76	194	2,634	74	156	2,459
	IR OPV maize	74	205	3,613	72	168	3,212
	STR hybrid maize	71	211	4,055	72	169	3,829
	WH403	70	191	3,998	70	157	3,311
	SE	1.4	4.7	329	0.8	3.5	282
Maize as sole crop	IR hybrid maize	78	183	2,006	74	157	2,104
	IR OPV maize	76	187	3,059	72	157	2,719
	STR hybrid maize	72	204	3,778	72	162	3,316
	WH403	70	184	3,715	71	162	3,249
	SE	1.8	4.9	411	0.6	157	281
Soybean rotation	IR hybrid maize	74	197	2,938	76	177	2,799
	IR OPV maize	73	199	3,503	74	173	3,033
	STR hybrid maize	67	224	4,457	73	185	3,396
	WH403	67	184	3,788	73	163	2,420
	SE	1.9	8.4	316	0.7	4.6	205

a Cropping system (main plot): Desmodium spp.; maize only; peanut; soybean rotation.

b Variety (subplot): IR HYB, imazpyr-resistant hybrid; IR OPV, imidazolinone-resistant open-pollinated variety; STR HYB, S. hermonthica-resistant hybrid; WH403, commercial maize hybrid.

^c^Abbreviations: CV, coefficient of variation; SD, significant difference.

Under artificial infestation, the IR maize hybrid and IR OPV had higher days to anthesis than other varieties across all cropping systems. However, days to anthesis were higher under *D. uncinatum* and maize only than in the peanut and soybean rotation. Therefore, combining (intercropping) IR maize varieties and *D. uncinatum* had less stress on the maize crop, resulting in increased days to anthesis.

Maize plants grown under the soybean–STR hybrid combination were the tallest; the peanut–IR hybrid had the shortest plants under natural infestation. Grain yield was high for the STR hybrid across all cropping systems. The peanut–STR hybrid combination yielded well, followed by the *D. uncinatum*‒ commercial hybrid WH403 combination under natural infestation. The STR–hybrid and WH403 benefited from the soil fertility enhancement effects of peanut and *D. uncinatum*.

IR maize varieties treated with imazapyr were very effective in controlling *S. hermonthica* across all the cropping systems at 8, 10, and 12 WAP. In the soil, imazapyr gradually dissipates and leaches down the profile, making it less available for *S. hermonthica* control with time. This would explain the increased *S. hermonthica* emergence with time from 8 to 12 WAP. Combining herbicide seed treatment of IR varieties with inter-cropping with peanut, D. uncinatum, and soybean rotation is a more effective method for *S. hermonthica* control than individual component technologies. Herbicide-based *Striga* spp. control early in the season favors crop establishment, and intercropping with a legume favors more long-term *Striga* spp. control. Growing maize in association with legumes (soybean, peanut, and D. uncinatum) and herbicide-resistant maize in the field resulted in lower emergence of *S. hermonthica*, hence better growth and yield of maize (Odhiambo et al. 2011), demonstrating the effectiveness of the intercrops in controlling *S. hermonthica* and reducing the seedbank. With the IR maize technology management option, *Striga* spp. germination is delayed and its ability to flower and set seed is reduced. This would eventually lead to a reduction in the *Striga* spp. seedbank. A combination of IR maize intercropped with legumes that also minimize *Striga* spp. seed setting should go a long way in reducing the parasite problem in smallholder farms in Africa.

Intercropping maize and beans is the most common cropping system in regions of Kenya where *S. hermonthica* is endemic. Working in western Kenya, Odhiambo and Ariga (2001) found intercropping maize and beans reduced S. *hermonthica* incidence and increased maize grain yields. Similar findings were reported by Kureh et al. (2006) in Nigeria and Odhiambo et al. (2011) in Kenya while growing maize in association with soybean. Intercropping and rotation resulted in a lower incidence of *S. hermonthica* and better growth and yield of associated maize. The high yield of maize in the intercrops and rotations show the possibility of realizing higher yields by optimizing benefits from the system. Soybean was more effective in reducing *S. hermonthica* infestation and gave a higher maize grain yield than cowpea (Kureh et al. 2006). Therefore, farmers should be encouraged to include grain legumes into the cereal cropping systems. Including legumes in a continuous maize system should result in increased maize production, improved soil fertility, and increased nutrient cycling (Bünemann et al. 2004). Therefore, where limited farm size and the need to produce maize every season preclude rotation with legumes, farmers should be encouraged to intercrop maize with legumes.

The results of this study showed that herbicide-treated IR hybrid and IR OPV maize were effective for S. *hermonthica* control. The STR hybrid containing *Striga* spp. resistance genes did not suffer from drastic yield losses in *S. hermonthica*-infested fields under both artificial and natural *S. hermonthica* infestations. The combination of herbicide seed treatment and genetic resistance to *Striga* spp. in a legume intercrop would serve as an effective integrated approach that would reduce both the parasite seedbank and the production of new *Striga* spp. seeds. These findings demonstrate that these technologies are effective in controlling the *S. hermonthica* weed with concomitant yield increases. They thus provide an opportunity to improve food security, stimulate economic growth, and alleviate poverty in the region. Farmers need to be encouraged to adopt the integration of crop management with herbicide-resistant maize for effective *Striga* spp. control.
